# Validation of a Novel, Sensitive, and Specific Urine-Based Test for Recurrence Surveillance of Patients With Non-Muscle-Invasive Bladder Cancer in a Comprehensive Multicenter Study

**DOI:** 10.3389/fgene.2019.01237

**Published:** 2019-12-18

**Authors:** Rui Batista, João Vinagre, Hugo Prazeres, Cristina Sampaio, Pedro Peralta, Paulo Conceição, Amílcar Sismeiro, Ricardo Leão, Andreia Gomes, Frederico Furriel, Carlos Oliveira, João Nuno Torres, Pedro Eufrásio, Paulo Azinhais, Fábio Almeida, Edwin Romero Gonzalez, Bohdan Bidovanets, Thorsten Ecke, Pascal Stinjs, Álvaro Serrano Pascual, Rabehi Abdelmalek, Ainara Villafruela, Pastora Beardo-Villar, Nuno Fidalgo, Hakan Öztürk, Carmen Gonzalez-Enguita, Juan Monzo, Tomé Lopes, Mario Álvarez-Maestro, Patricia Parra Servan, Santiago Moreno Perez De La Cruz, Mario Pual Sanchez Perez, Valdemar Máximo, Paula Soares

**Affiliations:** ^1^i3S—Instituto de Investigação e Inovação em Saúde, Porto, Portugal; ^2^Institute of Molecular Pathology and Immunology of the University of Porto (IPATIMUP), Porto, Portugal; ^3^U-Monitor Lda, Porto, Portugal; ^4^Faculty of Medicine, University of Porto, Porto, Portugal; ^5^Department of Pathology, Faculty of Medicine, University of Porto, Porto, Portugal; ^6^Department of Molecular Pathology, Portuguese Institute of Oncology, Coimbra, Portugal; ^7^Department of Urology Service, Portuguese Institute of Oncology, Coimbra, Portugal; ^8^Department of Urology, Hospital de Braga, Braga, Portugal; ^9^Department of Urology, Hospital CUF Coimbra, Coimbra, Portugal; ^10^Department of Urology, Hospital Universitário Fernando Pessoa, Porto, Portugal; ^11^Department of Urology, Hospital Zafra-Llerena, Badajoz, Spain; ^12^Department of Surgery, Ternopil Regional Oncology Center, Ternopil, Ukraine; ^13^Department of Urology, Helios Hospital, Bad Saarow, Germany; ^14^Department of Urology, St. Antonius Hospital, Nieuwegein, Netherlands; ^15^Servicio de Urología, Hospital Clínico San Carlos, Madrid, Spain; ^16^Independent Researcher, El-Eulma, Algeria; ^17^Department of Urology, Hospital Universitario Donostia, San Sebastian, Spain; ^18^Department of Urology, Hospital Universitario de Araba, Vitória, Spain; ^19^Department of Urology, Hospital Garcia de Horta, Lisbon, Portugal; ^20^Department of Urology, Medicalpark Izmir Hospital, Izmir, Turkey; ^21^Department of Urology, Hospital Universitario Fundacion Jiménez Díaz, Madrid, Spain; ^22^Department of Urology, Hospital de Santa Maria, Lisbon, Portugal; ^23^Department of Urology, Hospital Universitário La Paz, Madrid, Spain; ^24^Department of Urology, Hospital de Mérida, Badajoz, Spain; ^25^Department of Urology, Hospital Don Benito-Villanueva, Badajoz, Spain; ^26^Department of Urology, Hospital Universitário de Badajoz, Badajoz, Spain

**Keywords:** non-muscle invasive bladder cancer, TERT promoter mutation, FGFR3 mutation, urinary test, Uromonitor®

## Abstract

Bladder cancer (BC), the most frequent malignancy of the urinary system, is ranked the sixth most prevalent cancer worldwide. Of all newly diagnosed patients with BC, 70–75% will present disease confined to the mucosa or submucosa, the non-muscle-invasive BC (NMIBC) subtype. Of those, approximately 70% will recur after transurethral resection (TUR). Due to high rate of recurrence, patients are submitted to an intensive follow-up program maintained throughout many years, or even throughout life, resulting in an expensive follow-up, with cystoscopy being the most cost-effective procedure for NMIBC screening. Currently, the gold standard procedure for detection and follow-up of NMIBC is based on the association of cystoscopy and urine cytology. As cystoscopy is a very invasive approach, over the years, many different noninvasive assays (both based in serum and urine samples) have been developed in order to search genetic and protein alterations related to the development, progression, and recurrence of BC. *TERT* promoter mutations and *FGFR3* hotspot mutations are the most frequent somatic alterations in BC and constitute the most reliable biomarkers for BC. Based on these, we developed an ultra-sensitive, urine-based assay called Uromonitor^®^, capable of detecting trace amounts of *TERT* promoter (c.1-124C > T and c.1-146C > T) and *FGFR3* (p.R248C and p.S249C) hotspot mutations, in tumor cells exfoliated to urine samples. Cells present in urine were concentrated by the filtration of urine through filters where tumor cells are trapped and stored until analysis, presenting long-term stability. Detection of the alterations was achieved through a custom-made, robust, and highly sensitive multiplex competitive allele-specific discrimination PCR allowing clear interpretation of results. In this study, we validate a test for NMIBC recurrence detection, using for technical validation a total of 331 urine samples and 41 formalin-fixed paraffin-embedded tissues of the primary tumor and recurrence lesions from a large cluster of urology centers. In the clinical validation, we used 185 samples to assess sensitivity/specificity in the detection of NMIBC recurrence vs. cystoscopy/cytology and in a smaller cohort its potential as a primary diagnostic tool for NMIBC. Our results show this test to be highly sensitive (73.5%) and specific (93.2%) in detecting recurrence of BC in patients under surveillance of NMIBC.

## Introduction

Bladder cancer is the most frequent malignancy involving the urinary system and affects approximately four times more males than females (Miyazaki and Nishiyama, 2017). Worldwide, bladder cancer is the sixth most diagnosed cancer in men; when considering both genders, it ranks the 10th most diagnosed and the sixth position in prevalence (Ferlay et al., 2018; Ferlay et al., 2019). Of all patients newly diagnosed with bladder cancer, around three quarters present disease confined to the mucosa or submucosa (Sanli et al., 2017), the so-called, non-muscle-invasive bladder cancer (NMIBC) subtype (Babjuk et al., 2018). The remaining are classified as muscle invasive bladder cancer (MIBC), reflecting their capacity to infiltrate the muscle layer of the bladder (Alfred Witjes et al., 2017; Sanli et al., 2017). The current treatment for NIMBC is transurethral resection (TUR); following TUR treatment, 70% of the NMIBC patients will recur after primary tumor removal and 10–20% will recur as MIBC, with the capacity to progress and develop metastatic disease (Kaufman et al., 2009; van der Heijden, 2009; Chamie et al., 2013). This high rate of recurrence requires that patients are submitted to an intensive follow-up program. Major guidelines from the European Association of Urology (EAU) and American Urological Association (AUA) recommend cystoscopy and urinary cytology that, depending from the grade, can be as often as every 3 months in the first 2 years, semi-annually during the subsequent 3 years, and annually thereafter (Kassouf et al., 2016; Alfred Witjes et al., 2017; Babjuk et al., 2018). This intensive follow-up is maintained throughout many years following the initial diagnosis and indicates bladder cancer as a type of cancer with the most expensive follow-up (Kamat et al., 2011; Yeung et al., 2014). Cystoscopy is invasive and uncomfortable for patients due to the technical requirements of the procedure; still, it renders the more accurate diagnosis method for bladder cancer (Geavlete et al., 2012). Contrary to cystoscopy, noninvasive urine cytology is an economical approach, easier to perform, and when high-grade tumors are considered, the sensitivity is high (84%). The major limitation of urine cytology is its overall sensitivity to detect low-grade tumors (NMIBC), where the sensitivity decreases to 16%, precluding its use in the detection of those lesions (Yafi et al., 2015). The combination of all these facts leads to the opportunity for developing new, alternative, and minimally invasive methods to detect bladder cancer. As urine is in direct contact with the inner part of the bladder, cells from the epithelium, including scammed cells from bladder tumors, can exfoliate and be detected in urine and used to evaluate and monitor the presence of neoplasia in a noninvasive approach (Botezatu et al., 2000; Zwarthoff, 2008; Ralla et al., 2014; Critelli et al., 2016; Togneri et al., 2016). Over the years, many different noninvasive assays have been developed in order to search genetic and protein alterations known to be involved in the development, progression, and recurrence of bladder cancer, both in serum and urine samples, with the purpose to diagnose and monitor bladder cancer (Soloway et al., 1996; Fradet and Lockhard, 1997; Pode et al., 1999; Kruger et al., 2003; Tetu et al., 2005; Moonen et al., 2007; Halling and Kipp, 2008; Serizawa et al., 2011; Goodison et al., 2012; Kinde et al., 2012; Kinde et al., 2013; Allory et al., 2014; Bansal et al., 2014; Hurst et al., 2014; Ralla et al., 2014; Wang et al., 2014; Ellinger et al., 2015; Yafi et al., 2015; Springer et al., 2018; Miyake et al., 2018). Some of these tests presented values of sensitivity and specificity higher than urinary cytology and achieved FDA approval for bladder cancer diagnosis. Despite high sensitivities and specificities, all these molecular assays present inconvenient rates of false-positive results (Hajdinjak, 2008; Dimashkieh et al., 2013; Gopalakrishna et al., 2017; Springer et al., 2018). False-positive rates could result from several factors, including the presence of benign conditions as hematuria, cystitis, lithiasis, urinary tract infections, and inflammation or even because of repeated instrumentation, such as cystoscopy (Parker and Spiess, 2011; Dal Moro et al., 2013). A meta-analysis about the performance of urinary biomarkers concluded that most of the available urinary biomarkers do not detect the presence of bladder cancer in a proportion of patients and allow false-positive results in others, more frequently in low-stage and low-grade tumors (Chou et al., 2015). So, more reliable biomarkers and assays are needed for earlier detection of bladder cancer recurrence, particularly in low-grade and low-stage NMIBC. Long non-coding RNAs (lncRNAs) have emerged as potential biomarkers since aberrant expression has been reported in bladder cancer, some upregulated (lncRNA urothelial cancer associated 1 and lncRNA metastasis-associated lung adenocarcinoma transcript 1) (Wang et al., 2008; Han et al., 2013), and others downregulated such as maternally expressed 3 (MEG3) (Ying et al., 2013). Some new potential therapeutic targets were also described, such as MIR503 host gene (MIR503HG) and lncRNA MALAT-1 (Ying et al., 2012; Qiu et al., 2019). Recently, telomerase reverse transcriptase (*TERT*) promoter methylation aberration has been found in a large number of cancers, in a region described as *TERT* hypermethylated oncological region (THOR). THOR hypermethylation has been found as an alternative telomerase-activating mechanism in cancer that can act independently or in conjunction with *TERT* promoter mutations, further supporting the utility of THOR hypermethylation as a prognostic biomarker (Lee et al., 2018). Other studies highlight that both THOR hypermethylation and *TERT* promoter mutations are common and coexist in bladder cancer, and while *TERT* promoter mutation behaves as an early event in bladder carcinogenesis, THOR hypermethylation seems associated with disease progression, with the combined genetic and epigenetic alterations of *TERT* bringing additional prognostic value in NMIBC (Leão et al., 2019). *TERT* promoter mutations *per se* emerged as a novel biomarker detected in up to 80% of bladder cancer, independently of stage or grade (Rachakonda et al., 2013; Allory et al., 2014; Hurst et al., 2014; Hosen et al., 2015). *TERT* promoter (*TERTp*) mutations are the most common event across stages and grades in malignant bladder tumors, strongly suggesting its participation in the two major genetic pathways of urothelial tumorigenesis (Allory et al., 2014; Hurst et al., 2014). These features point *TERTp* mutations as a game changer in bladder cancer and pointed them to be considered as a useful urinary biomarker for disease monitoring and early detection of recurrence, even in low-grade NMIBC, where urinary cytology usually lacks sensitivity (Allory et al., 2014; Hurst et al., 2014; Vinagre et al., 2014; Descotes et al., 2017). *TERTp* mutations are not present in inflammatory or urinary infections, different from previously described urinary biomarkers (Raitanen et al., 2001; Chou et al., 2015; Descotes et al., 2017). *TERTp* mutations assumed a novel pivotal role, even surpassing the frequency of the oncogene-activating mutations in fibroblast growth factor receptor 3 (*FGFR3*) gene in NMIBC (Netto, 2011; Humphrey et al., 2016), one of the most relevant drivers of urothelial transformation. Cappellen et al. reported *FGFR3* mutations in bladder cancer with a frequency of 35%, and subsequent studies established this frequency in approximately half of the primary bladder tumors (Cappellen et al., 1999; Sibley et al., 2001). Several studies report its presence in up to 80% regarding early-stage and low-grade tumors and as absent or a very rare event in high-grade and invasive tumors (Billerey et al., 2001; van Rhijn et al., 2003; Hernandez et al., 2006; Tomlinson et al., 2007; Pandith et al., 2013). *FGFR3* assumes also an important role as a predictive biomarker due to the development of *FGFR3*-targeted therapies. *KRAS* mutations, although found in a lower percentage (11.5%) of bladder cancers, are assuming a relevant position since the detection of *KRAS* mutations in conjunction with the previous alterations could improve the sensitivity of a biomarker panel (Alexander et al., 2012).

The uniqueness of *TERTp* mutations, mainly its location in a promoter region with a GC base pair content >50% precluded that traditional methods using standardized conditions (conventional real-time assays or even next-generation sequencing techniques) could be used with an efficient output. With this goal in mind, we developed an ultra-sensitive assay based on real-time PCR (with a proprietary reaction chemistry and probes), a urine-based test capable of detecting trace amounts of the most common alterations in NMIBC, *TERTp* c.1-124C > T, c.1-146C > T, and *FGFR3* p.R248C, p.S249C hotspot mutations, in urine samples.

## Material and Methods

All procedures described in this study were in accordance with national and institutional ethical standards and the Declaration of Helsinki. Written informed consent was obtained from the patients participating in the study. Procedures were previously approved by Ethical Review Committee IPO-Coimbra (03/TI/15). Inclusion and exclusion criteria for each analysis are detailed in **Supplementary Table 1**.

### Sample Collection

#### Urine Samples

Urine samples from each participating urology center were collected and processed for delivery during routine urology appointments and previously to cystoscopy intervention, according to Uromonitor recommendations. Urine samples were filtered through a pretreated 0.80-µm nitrocellulose syringe filter (Whatman^®^ Filter—Z612545, Merck, Germany) containing a homemade conservative storage buffer (10 mM glutathione, 1 M lithium chloride–6 M urea–30 mM Biuret, 2 M EDTA (E7889-100ML) (information on this process is available in the video on the **Supplementary—Video 1**). Filters were then sent to the central lab at Ipatimup/I3S (Porto, Portugal). After arrival, filters were stored at 2–8°C for a maximum of 1 month until DNA extraction procedure.

#### Tissue Samples

Formalin-fixed paraffin-embedded (FFPE) tissues from primary tumor and/or from recurrent lesions from the cohort in study were obtained from the repository of tumors of the Instituto Português de Oncologia de Coimbra Francisco Gentil, E.P.E (IPOC-FG). Clinicopathological and follow-up data were retrieved from the files of the Department of Pathology of IPOC-FG.

### Cohort’s Characteristics—Urine and FFPE Cohorts

We studied a total of 372 samples (331 urine samples and 41 FFPE) collected from 18 urology centers (**Supplementary Table 2**). Technical validation of the assay was done in an independent setting where we studied a total of 334 samples from urine and FFPE (presented below). Clinicopathological and follow-up data were retrieved from the files of the centers involved in this study (**Supplementary Figures 1** and **2**).

The main aim of our study was to validate our new molecular panel in samples obtained through noninvasive procedures. For this, we performed technical validation by analyzing a total of 331 urine samples. The clinical validation was performed by accessing the test’s ability to correctly detect patients who do have the condition, by calculating the ratio between the number of the test true positives and the total number of patients that harbor active disease (sensitivity). Also, specificity, the test’s ability to correctly reject healthy patients without a condition, was calculated with the ratio between the number of true negatives obtained by the test and the total number of patients that do not harbor disease at the time of the test. Positive predictive value (PPV) (ratio between test true positives and all the test positives) and negative predictive value (NPV) (ratio between test true negatives and all the test negatives) were also calculated, aiming at knowing after the test result, the probability that the patient has (or does not have) the disease. All these calculations were done using data from 185 patients.

Thus, urine samples from 185 patients (77% males and 33% females), with a median age of 71 years (range, 25–91) **Table 1**, were used and were subdivided into independent groups (that may overlap samples) (**Table 2**).

**Table 1:** Clinical validation cases information and clinicopathological data.

	Characteristics	Total cases (*n* = 185)
Age (years)		
	Median age (range)	71 (25–91)
		
Age cluster, *n* (%)		
	20–39	9 (4.9)
	40–59	39 (21.3)
	60–79	102 (55.7)
	80+	33 (18.0)
Gender, *n* (%)		
	Female	41 (23.2)
	Male	136 (76.8)
Smoking status, *n* (%)		
	Yes/Former	45 (39.5)
	No	69 (60.5)
Disease status, *n* (%)		
	Primary	122 (65.9)
	Recurrence	63 (34.1)
Stage, *n* (%)		
	Cis/Tis	5 (9.8)
	Ta	32 (62.7)
	T1	12 (23.5)
	T2	1 (2)
	Hep.Met	1 (2)
Grade, *n* (%)		
	Low grade	25 (51)
	High grade	24 (49)
Urine cytology, *n* (%)		
	Positive/atypical cytology	12 (14.3)
	Negative cytology	72 (85.7)
Cystoscopy, *n* (%)		
	Positive	65 (35.2)
	Negative	120 (64.8)

**Table 2:** Cohorts used in this study.

Cohort name	Cohort designation	No. of samples
**Follow-up cohort**	Urine samples from patients under follow-up for NMIBC	122
**Initial diagnosis cohort**	Urine samples from patients screened for bladder cancer	63
**Tumor samples cohort**	FFPE samples from primary tumors and recurrence from patients under follow-up for NMIBC	41
**Uromonitor + ** ***KRAS*** ** follow-up cohort**	Urine samples from patients under follow-up for NMIBC screened for both Uromonitor^®^ and *KRAS* hotspot alterations	24
**Uromonitor + ** ***KRAS*** ** initial diagnosis cohort**	Urine samples from patients screened for bladder cancer for both Uromonitor^®^ and *KRAS* hotspot alterations	25

FFPE tissues of the primary tumor (*n* = 9) and/or of the recurrence lesions (*n* = 32) were also analyzed to test the performance of this assay in a different biological sample (FFPE) other than urine.

#### Cystoscopy, Cytology, and Tumor Resected Evaluation

Cystoscopy was considered positive when an unequivocal lesion deserving surgical treatment (despite the pathology result of the resected lesion) was observed by the urologist. Urine cytology and tissue pathology were performed by each pathology department from each center. In 41 cases, the diagnosis was confirmed in the histological examination of the lesion in the TUR.

### DNA Extraction

#### Urine Samples

Filters used for urine filtration were stabilized at room temperature for 30 min. Upon filtration, quality DNA for further processing is obtained on filters that can be stored at 4°C, up to 3 months (**Supplementary Figure 3**). In an inverted position, filters were attached to a 2-ml microcentrifuge tube and a cell lysis solution was injected through each filter and collected in a microcentrifuge tube. Filtered lysates were incubated at 60°C for 30 min with 30 µl of proteinase K at 10 mg/ml, exposed to chaotropic lysis/binding buffer (Citogene Cell Lysis Buffer, Citomed, Portugal) to release nucleic acids and protect the genomic DNA from DNases. The microcentrifuge tube content was then processed according to the manufacturer’s protocol of the Norgen^®^ Plasma/Serum Cell-Free Circulating DNA Purification Mini Kit (Norgen Biotek Corp, Canada).

#### Tissue Samples

DNA from FFPE tissues was retrieved from 10-µm cuts after careful manual dissection. Slides were deparaffinized in xylene (2 × 10 min), followed by incubation in 100% alcohol (2 × 5 min). Tumor tissue was removed from the slides into a 1.5-ml microcentrifuge tube. DNA extraction was performed using the Ultraprep Tissue DNA Kit (AHN Biotechnologie, Germany) according to the manufacturer’s instructions. The DNA extracted was quantified by spectrophotometry using Nanodrop ND-1000, and quality was assessed by analysis of 260/280 and 260/230-nm ratios.

### Urine Testing Workflow

**Figure 1:**
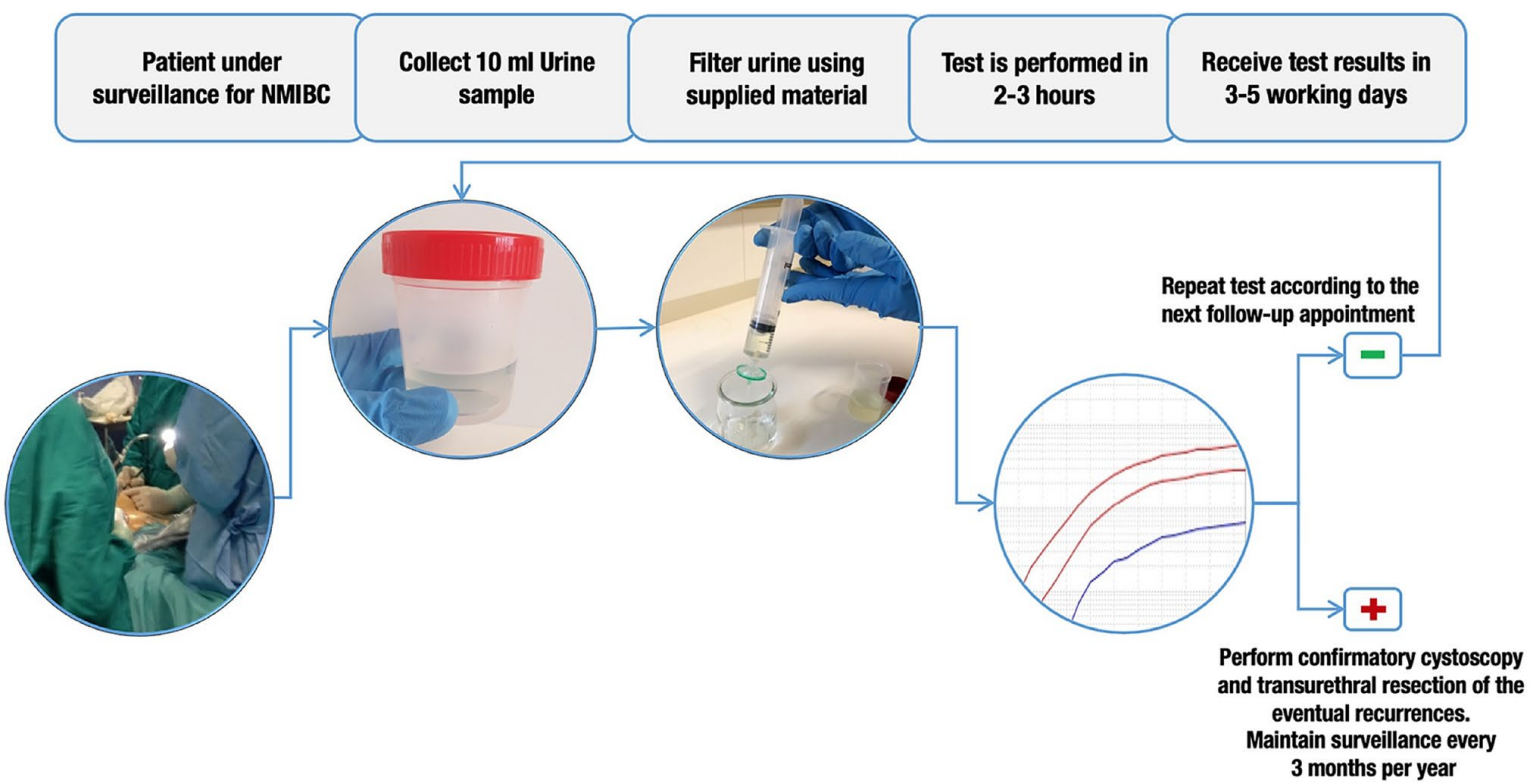
Urine testing workflow. In patients under surveillance for non-muscle-invasive bladder cancer (NMIBC), a minimum of 10 ml of urine is collected before cystoscopy. This 10 ml of urine is then filtered through a 0.8-µm filter and stored at 4°C. DNA extraction and Uromonitor^®^ test are then performed. If a positive result is obtained, confirmatory cystoscopy and transurethral resection of eventual recurrences are recommended. If a negative result is obtained, it is recommended that the test should be repeated on next follow-up appointment.

The Uromonitor^®^ is a custom-made full working procedure developed and optimized for the detection in a real-time PCR platform of oncogene hotspot mutations in bladder cancer tumor cells, exfoliated to urine, particularly *TERTp* c.1-124C > T, *TERTp* c.1-146C > T, *FGFR3* p.R248C, and *FGFR3* p.S249C alterations (**Figure 1**). All the tests done in this study were performed in the central lab at Ipatimup/I3S by the same lab professional, ensuring minor variability in data creation and analysis.

**Figure 2:**
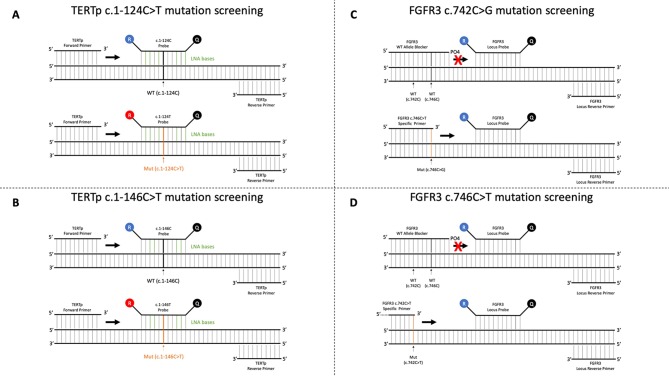
Technical principles of the test. **(A)** Real-time qualitative method optimized for *TERTp* c.1-124C > T detection. Two competitive fluorescent probes targeting normal (WT- c.1-124C) and mutated (Mut- c.1-124C > T) alleles incorporating Locked Nucleic Acid bases are used to detect the mutations. **(B)** Real-time qualitative method optimized for *TERTp* c.1-146C > T detection. Two competitive fluorescent probes targeting normal (WT- c.1-146C) and mutated (Mut c.1-146C > T) alleles incorporating Locked Nucleic Acid bases are used to detect the mutations. **(C)** Real-time qualitative method optimized for *FGFR3* c.742C > G detection. A mutation allele-specific primer, a phosphorylated wild-type allele blocker that completely suppresses the amplification of the wild-type allele, a locus reverse primer, and a fluorescent probe for real-time detection of the generated amplicon are used. **(D)** Real-time qualitative method optimized for *FGFR3* c.746C > T detection. A mutation allele-specific primer, a phosphorylated wild-type allele blocker that completely suppresses the amplification of the wild-type allele, a locus reverse primer, and a fluorescent probe for real-time detection of the generated amplicon are used.

Mutation detection is achieved by real-time PCR amplification curve analysis. Positive and negative mutation control samples are included in each run to ensure the assay’s validity. For *TERTp* c.1-124C > T and c.1-146C > T alteration screening, we developed an improved real-time allelic discrimination assay (further referred to in the text as *TERT*-124 and *TERT*-146 assays), with the use of Locked Nucleic Acid (LNA probes) (**Figures 2A, B**). LNA probes allowed modulating the melting temperature on specific bases of the probe, enhancing the possibility to achieve preferential melting temperatures in short probe sequences. LNA probes greatly improve allelic discrimination, allowing higher stability on the binding to a specific target even with a shorter sequence. When in the presence of a base pair mismatch, such specificity is lost due to a large melting temperature difference. This leads to the impossibility for the LNA probe to bind to a sequence that contains only one base pair mismatch. This high specificity to the target sequence renders this type of probe perfect for allelic discrimination experiments (**Supplementary Figure 4**).

For *FGFR3* mutations selected for screening (p.R248C and p.S249C), we designed for each mutation a competitive allele-specific real-time detection PCR (further referred to in the text as *FGFR3* 248 and *FGFR3* 249 assays), based on the design of a mutation allele primer, a wild-type allele blocker, a locus reverse primer, and a fluorescent probe for real-time detection of the generated amplicon (**Figures 2C, D**). The use of a molecular blocker suppressed the amplification of the wild-type allele in order to not interfere with the amplification of the mutant allele. By this technique, we improved current detection limit for the selected alterations compared to Sanger sequencing, enhancing the ability to detect a minimal quantity of altered cells in a large pool of cells without alterations.

In this work, we also present preliminary results on the high-sensitivity screening of *KRAS* codon 12 and codon 61 alterations achieved through the use of a custom-made mutation detection procedure developed similarly to *FGFR3* hotspot mutation detection procedure, rendering this method suitable for detection of mutations in bladder cancer tumor cells exfoliated to urine.

*TERT*, *FGFR3*, and preliminary *KRAS* testing was performed in approximately 25 ng of DNA extracted from cells in each filtered urine, or from 25 ng of DNA extracted from FFPE tissues, either primary tumor and/or recurrent lesions. The extracted DNA was amplified and detected on a qPCR real-time machine using the proprietary chemistry for amplification and detection as provided in the Uromonitor^®^ test kit for the targeted nucleotide changes in *TERTp* and *FGFR3* genes.

### Uromonitor^®^ Technical Validation

Uromonitor^®^ precision was analyzed by a reproducibility test. To achieve this, 10 samples were amplified and analyzed using Uromonitor^®^ test (eight samples harboring mutations and two wild-type samples for the alterations of interest). These samples were amplified five times for each alteration, 1 week apart of each amplification, for 5 weeks. Uromonitor^®^ accuracy was analyzed by two independent tests. First, it was necessary to ensure that a test containing a sample without DNA or with DNA that does not harbor any of the alterations of interest did not generate an analytical signal that may indicate a low concentration of mutation (analytical false positive). It was also necessary to assess the accuracy of the results produced by Uromonitor^®^ test comparing it to the standard method in the detection of the alterations in study (Sanger sequencing). All the samples were validated by Sanger sequencing for the alterations in study.

#### Uromonitor**^®^** Precision and Accuracy in Urine Samples

To test accuracy in urine samples, 36 samples negative for all the mutations in study (status obtained by Sanger sequencing) and 36 “blank” samples (without DNA) were amplified for each alteration (false-positive testing). 

Also, 252 blind tests from urine samples were analyzed (73 tests for *TERTp* −124 assay, 72 tests for *TERTp* −146 assay, 55 tests for *FGFR3* 248 assay, and 52 tests for *FGFR3* 249 assay).

#### Uromonitor**^®^** Precision and Accuracy in FFPE Tissue Samples

Uromonitor^®^ test could also be used to screen FFPE samples in patients with a history of NMIBC. To test accuracy in FFPE tissue samples, nine samples negative for all the mutations in study (status obtained by Sanger sequencing) and 36 “blank” samples (without DNA) were amplified for each alteration (false-positive testing). Also, 483 tests from FFPE tissue samples were analyzed (201 tests for *TERTp* −124 assay, 200 tests for *TERTp* −146 assay, 41 tests for *FGFR3* 248 assay, and 41 tests for *FGFR3* 249 assay).

#### ***TERTp*** Detection Limit Assessment

**Figure 3:**
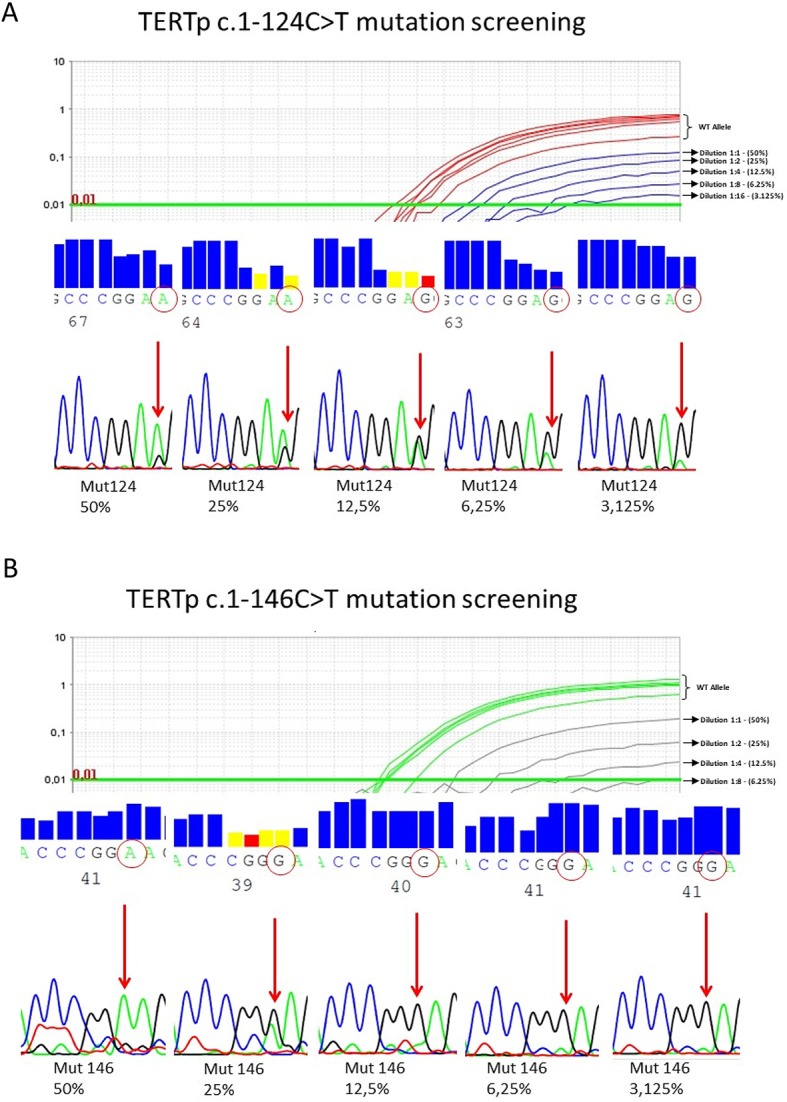
**(A)** Serial dilution detection of *TERTp* c.1-124C > T. *TERTp* c.1-124C > T mutated DNA with 50% (wild-type) WT/mutation ratio was diluted in a twofold dilution (eight dilutions) in WT DNA. Detection limit was fixed at the presence of 6.25% of *TERTp* c.1-124C > T alteration in the total DNA in a reaction with 25 ng of total DNA. Below this limit, mutation detection is not guaranteed. **(B)** Serial dilution detection of *TERTp* c.-146C > T. *TERTp* c.1-146C > T mutated DNA with 50% WT/mutation ratio was diluted in a twofold dilution (eight dilutions) in WT DNA. Detection limit was fixed at the presence of 6.25% of *TERTp* c.1-146C > T alteration in the total DNA in a reaction with 25 ng of total DNA. Below this limit, mutation detection is not guaranteed.

Uromonitor^®^ includes *TERTp* alteration detection by real-time PCR by LNA allelic discrimination probes. High GC content and thorough optimization of the amplified *TERTp* alterations characterize this innovative test. Since *TERTp* mutation detection by current methods has low sensitivity, there was the need to assess the detection limit for *TERTp* alterations included by the technology in the Uromonitor^®^. To achieve this, we performed twofold serial dilutions of genomic DNA containing the studied alteration (100% of mutated DNA) in genomic DNA wild type for the studied alterations. Serial dilutions were amplified for the corresponding detection assay. This procedure was repeated for both *TERTp* alterations that comprise the Uromonitor^®^ test (**Figure 3**).

#### Statistical Analysis

Statistical analysis was performed using 21.0 SPSS Statistical Package (SPSS, Inc., 220, 2003). Descriptive statistic was done and the results are expressed as percentages and mean ± standard deviation.

## Results

### Genetic Alterations Technical Validation

Uromonitor^®^ precision was analyzed, achieving a 100% concordance in a reproducibility test. In urine samples accuracy tests (comparisons to Sanger sequencing), *TERTp* −124 assay achieved 100%, *TERTp* −146 assay 98.6%, *FGFR3* 248 assay 87.3%, and *FGFR3* 249 assay 94.2%. Overall, Uromonitor^®^ test presented a combined accuracy of 95.0%. Uromonitor^®^ test accuracy in FFPE tissue samples (comparison to Sanger sequencing) achieved 98.5% for *TERTp* −124 assay, 99.5% for *TERTp* −146 assay, 90.2% for *FGFR3* 248 assay, and 97.6% for *FGFR3* 249. For all assays, Uromonitor^®^ achieved a combined 96.5% accuracy (**Supplementary Table 3**). The test presented no false positives in samples without DNA (blank samples). A combined accuracy lower than 100% is justified by the detection of positivity by real-time PCR in samples for which Sanger sequencing fails to detect alteration due to lack of sensitivity.

In all the assays, the analytical detection limit was 6.25% of mutant sequences in a background of wild-type DNA. The presence of altered DNA in less than 6.25% of the total DNA in the sample may not be detected.

#### Molecular Characterization of Urine Samples

From the initial cohort of 331 urine samples, 304 were fully characterized for the alterations targeted by Uromonitor^®^ test and 27 failed one or more alterations. From these, *TERTp* mutations were detected in 50.6% of cases (39.0% presented the *TERTp* c.1−124C > T and 11.7% with the *TERTp* c.1−146C > T) and *FGFR3* mutations were detected in 49.4% of cases (31.2% at codon 248 and 18.2% at codon 249 of *FGFR3* protein). Further correlations with clinical data were performed for 185 samples where complete clinical data was available.

### Clinical Validation

#### Recurrence Follow-Up Cohort

In the follow-up cohort (*n* = 122), 28% (*n* = 34) of the patients recurred (confirmed by histology) of the TUR, whereas the remaining 72% (*n* = 88) were negative for recurrence.

##### Uromonitor® Performance Comparison With Cytology and Cystoscopy Methods

We analyzed and compared follow-up recurrence detection of Uromonitor^®^ in NMIBC in comparison to routinely used screening methods such as cystoscopy and/or cytology.

**Figure 4:**
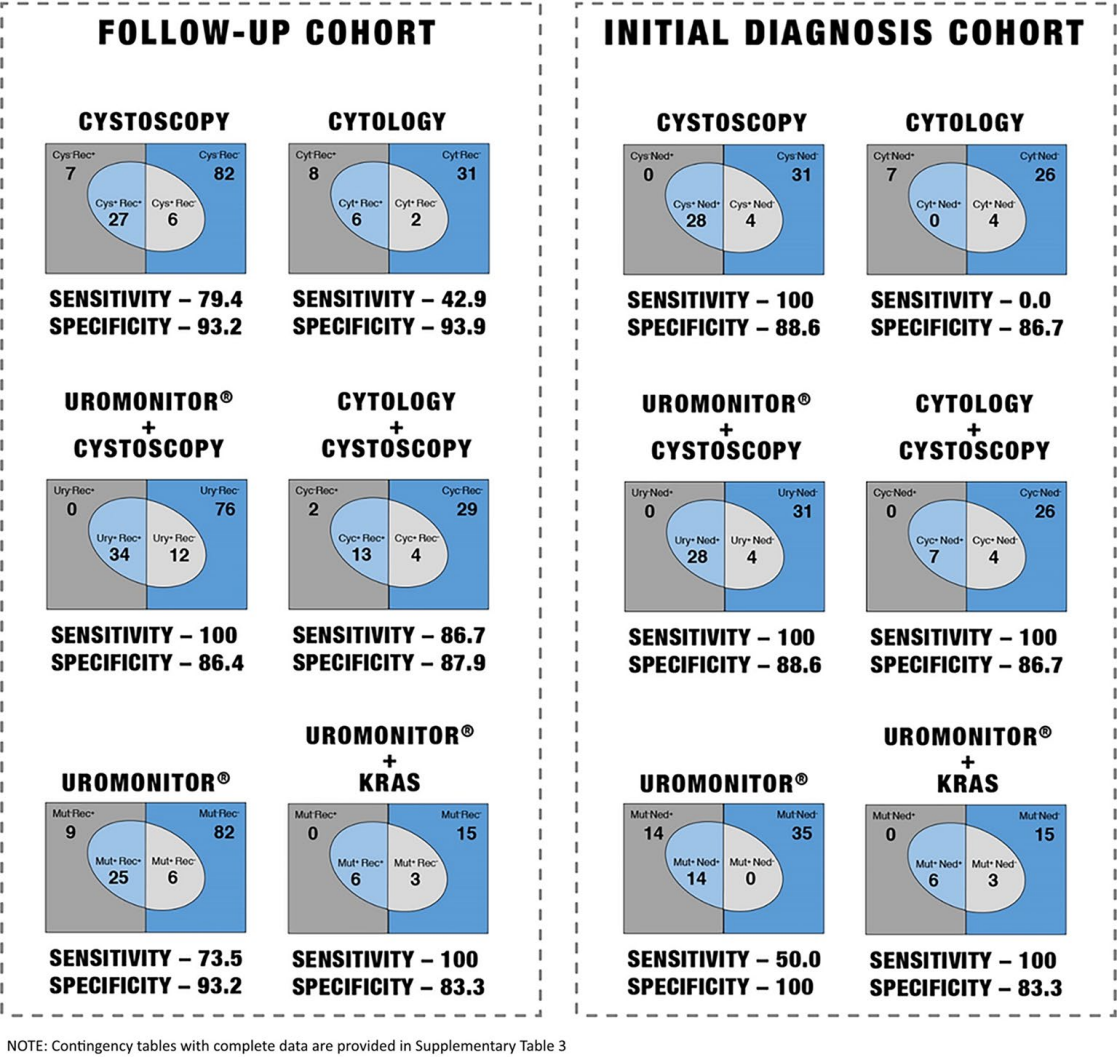
Performance of different screening methods in non-muscle-invasive bladder cancer (NMIBC) follow-up recurrence detection and in NMIBC diagnosis.

Uromonitor^®^ sensitivity was 73.5% in the detection of TUR confirmed recurrence, with a specificity of 73.2% (**Figure 4**, **Table 3** and **Supplementary Table 4**). The values were comparable and similar to gold-standard cystoscopy performance that in the follow-up series presented values of 79.4% and 73.2% for sensitivity and specificity, respectively (**Figure 4**, **Table 3** and **Supplementary Table 4**).

**Table 3:** Performance of different screening methods in non-muscle-invasive bladder cancer (NMIBC) follow-up recurrence detection and in NMIBC initial diagnosis.

	UROMONITOR	CYSTOSCOPY	Cytology	Cystoscopy + cytology	UROMONITOR + cystoscopy	UROMONITOR + *KRAS*
**Follow-up cohort**						
**Sensitivity**	50.0	79.4	42.9	86.7	100.0	100
**Specificity**	100.0	93.2	93.9	87.9	86.4	83.3
**Accuracy**	77.8	89.3	78.7	87.5	90.2	87.5
**PPV**	100.0	81.8	75.0	76.5	73.9	66.7
**NPV**	71.4	92.1	79.5	93.5	100.0	100
**Initial diagnosis cohort**						
**Sensitivity**	50.0	100.0	0.0	100.0	100.0	93.3
**Specificity**	100.0	88.6	86.7	86.7	88.6	80.0
**Accuracy**	77.8	93.7	70.3	89.2	93.7	88.0
**PPV**	100.0	87.5	0.0	63.6	87.5	87.5
**NPV**	71.4	100.0	78.8	100.0	100.0	88.9

Uromonitor^®^ sensitivity performance was much higher than cytology (42.9% cytology sensitivity vs. 73.5% Uromonitor^®^ sensitivity (**Figure 4**, **Table 3** and **Supplementary Table 4**).

When cytology was combined with cystoscopy, they jointly achieved an increased sensitivity of 86.7% and a slightly decreased specificity of 87.9% due to the increased rate of cytology false positives (**Figure 4**, **Table 3** and **Supplementary Table 4**). Although the combination with cytology presents an upgrade to cystoscopy *per se*, a greater benefit is obtained when combining Uromonitor^®^ with cystoscopy, granting together a 100% sensitivity and 88.6% specificity, clearly demonstrating an improvement in sensitivity and specificity relative to the “cystoscopy + cytology” screening method (**Figure 4**, **Table 3** and **Supplementary Table 4**).

To further improve Uromonitor^®^ test performance, we analyzed a subset of samples (Uromonitor^®^ + *KRAS* follow-up cohort) for another oncogene activated in bladder cancer, *KRAS* hotspot alterations, and compared follow-up recurrence detection to routinely used surveillance methods.

*TERT*/*FGFR3*/*KRAS* increased sensitivity to 100% in the detection of TUR confirmed recurrence with a specificity of 83.3% (**Figure 4**, **Table 3** and **Supplementary Table 4**). The values were higher when compared to cystoscopy performance that in the follow-up series achieved 79.4% and 73.2% for sensitivity and specificity, respectively (**Figure 4**, **Table 3** and **Supplementary Table 4**). *TERT*/*FGFR3*/*KRAS* sensitivity performed higher than cytology (42.9% sensitivity and 93.9% specificity) (**Figure 4**, **Table 3** and **Supplementary Table 4**).

Compared with cytology combined with cystoscopy, although this combination presents an interesting upgrade to cystoscopy *per se*, it does not achieve *TERT*/*FGFR3*/*KRAS* molecular testing performance *per se* or molecular testing in combination with cystoscopy screening method (**Figure 4**, **Table 3** and **Supplementary Table 4**).

#### Genetic Alterations Distribution in Recurrences and Initial Diagnostic Positive Cases

**Figure 5:**
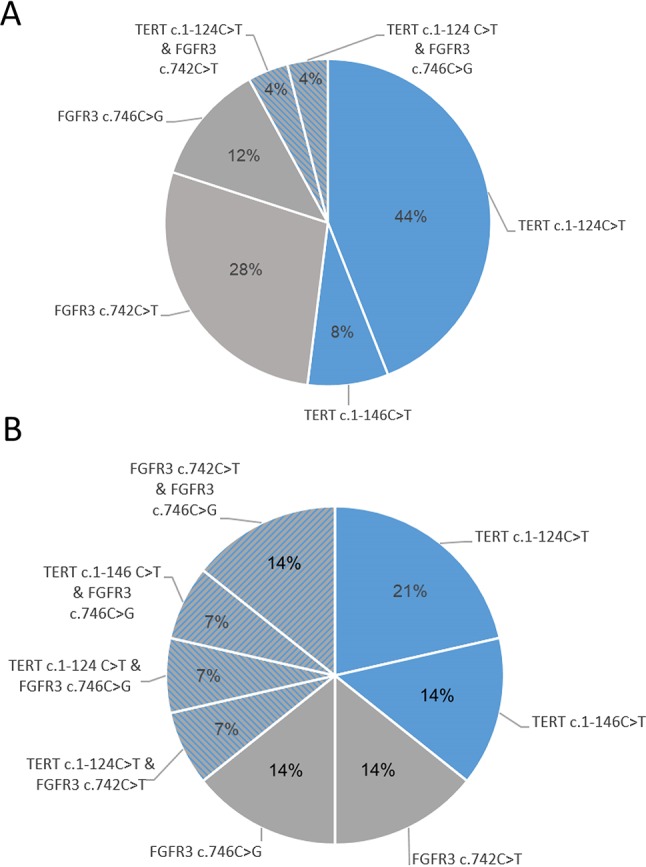
Mutation distribution across follow-up cohort **(A)** and initial-diagnosis cohort **(B)**.

Regarding the specific mutations detected in positive urine samples from the follow-up cohort, *TERTp* mutations were detected in 52.0% of cases (44.0% presented the −124C > T and 8.0% with the −146C > T) and *FGFR3* mutations were detected in 40.0% of cases (28.0% at codon 248 and 12.0% at codon 249 of *FGFR3* protein). Of the cases, 8.0% presented two concomitant alterations (two cases with *TERTp* c.1−124C > T and with alterations in codons 248 or 249 of *FGFR3*) (**Figure 5**).

Regarding the specific mutations detected in urine samples from the cohort of patients undergoing initial diagnosis, they were positive for Uromonitor assay as follows: *TERTp* mutations were detected in 35.7% of cases (21.4% presented the −124C > T and 14.3% the −146C > T mutation) and *FGFR3* mutations were detected in 28.6% of cases (14.3% at codon 248 and 14.3% at codon 249 of *FGFR3* protein). Of the cases, 35.7% presented *TERTp* and *FGFR3* concomitant alterations (one case with c.1-124C > T and p.R248C, one case with c.1-124C > T and p.S249C, one case with c.1-146C > T and p.S249C, and two cases with p.R248C and p.S249C alterations (**Figure 5**). Nine cases in the follow-up cohort and 14 cases in the initial diagnosis cohort did not present any of the *TERTp* or *FGFR3* screened alterations (**Figure 5**).

##### Uromonitor^®^ Performance Correlation With Stage/Grade

**Figure 6:**
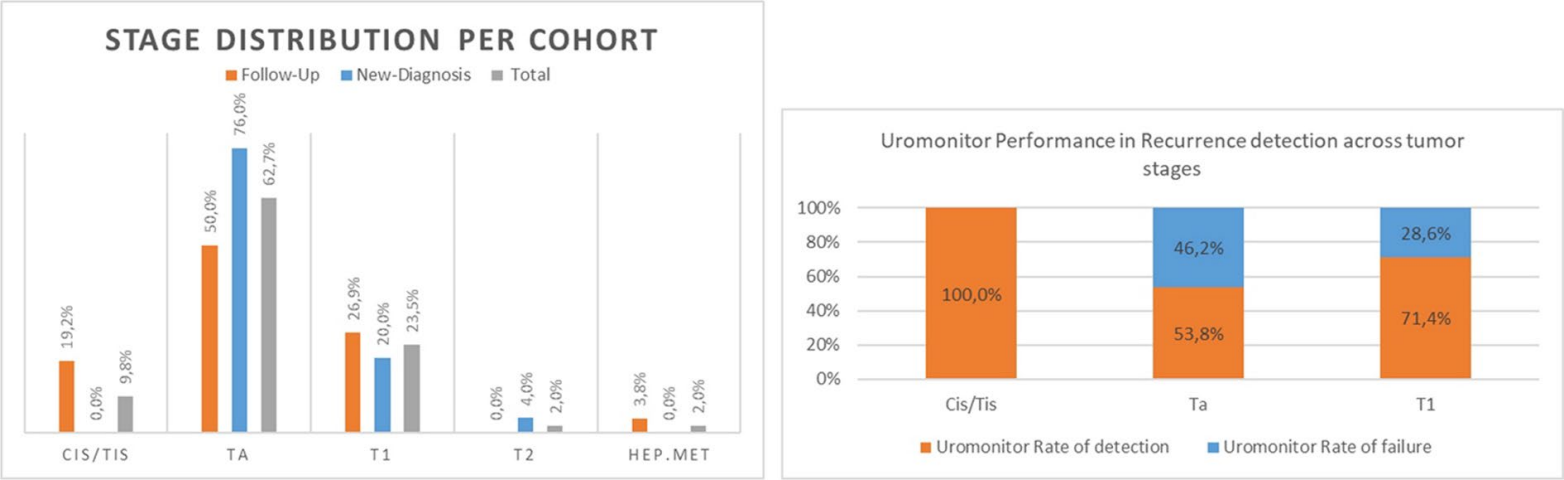
Cohort’s tumor stage distribution and Uromonitor performance in recurrence detection across tumor stages.

**Figure 7:**
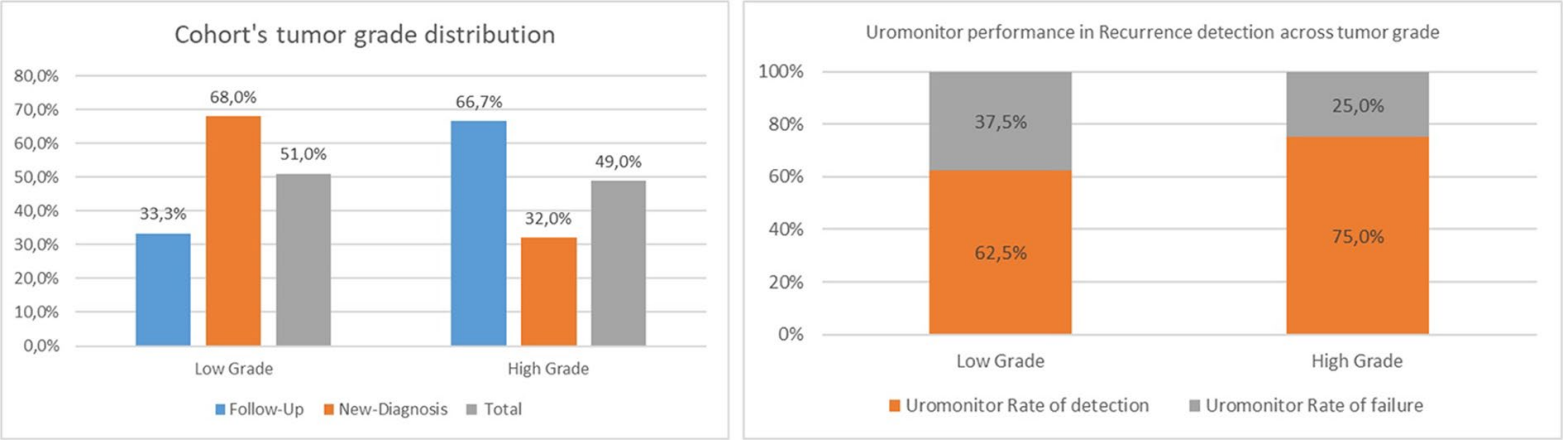
Cohort’s tumor grade and uromonitor performance in recurrence detection across tumor grades.

Tumor stage information in cases positive for recurrence was available for 26 patients. The majority of recurrence-positive cases were for stage Ta (50.0%), with T1 and Tis representing 27.0% and 19.2%, respectively (**Figure 6** and **Supplementary Table 5**). Regarding the grade, the majority of recurrence-positive tumors were high grade (66.7%), with 33.3% being the remaining low-grade cases (**Figure 7** and **Supplementary Table 6**). In Cis/Tis recurrence-positive patients, Uromonitor^®^ achieved a 100% detection rate, while in patients that recurred with a Ta tumor the detection rate was 53.8%. For T1 stage positive patients, the detection rate was 71.4% (**Figure 6** and **Supplementary Table 7**). In low-grade recurrence-positive patients, Uromonitor^®^ achieved a 62.5% detection rate, while in patients that recurred with a high grade, Uromonitor^®^ tumor detection rate was 75%. (**Figure 7** and **Supplementary Table 8**). One case (3.8%) presented a hepatic metastasis, positively detected in the urine sample by Uromonitor Uromonitor (**Figure 6** and **Supplementary Table 5**).

#### Initial Diagnosis Cohort Analysis

##### Uromonitor® Performance Comparison With Cystoscopy Method

We analyzed the diagnostic performance of the test in the initial diagnosis of bladder cancer in comparison to the usual screening methods such as cystoscopy and/or cytology.

Sensitivitywas 50.0% in an initial diagnosis setting, but with a specificity of 100%. These values are low in comparison with cystoscopy’s virtual sensitivity of 100%, although with a lower specificity of 88.6%, but are much better than cytology which did not have sensitivity (0%) and achieved 86.7% specificity.

We analyzed the performance of Uromonitor^®^ + *KRAS* in the initial diagnosis of bladder cancer in comparison to cystoscopy and/or cytology. Uromonitor^®^ + *KRAS* sensitivity was 93.3% in an initial diagnosis setting with a specificity of 80% (**Figure 4**, **Table 3**, and **Supplementary Table 4**).

## Discussion

*TERT* promoter mutations were firstly described in sporadic and familial melanoma (Horn et al., 2013; Huang et al., 2013), and since then they were reported in several cancers, such as central nervous system (43–51%), hepatocellular carcinoma (59%), thyroid (follicular cell-derived tumors) (10%), and notably in bladder cancer (59–80%) (Killela et al., 2013; Liu et al., 2013a; Liu et al., 2013b; Nault et al., 2013; Vinagre et al., 2013; Wu et al., 2014). For bladder cancer, the *TERTp* mutations are independent of stage or grade (Rachakonda et al., 2013; Allory et al., 2014; Hurst et al., 2014; Hosen et al., 2015) and were reported in both non-muscle and muscle-invasive bladder cancer. As the current diagnosis and follow-up of patients with bladder cancer is highly invasive and expensive, new molecular markers are needed able to act in noninvasive approaches in order to select an optimal treatment and follow-up for each patient (Kurth et al., 1995; Pandith et al., 2013; van Kessel et al., 2013). For this purpose, we developed a novel urine-based real-time assay (Uromonitor^®^), and in this study, we present the technical and clinical performance of the detection of critical alterations in *TERT p* region and *FGFR3* in DNA obtained from scammed cells of bladder present in urine. The main goal of the test is to be able to predict recurrence in NMIBC *per se* or in combination with cystoscopy. In this study, we analyzed and compared its performance in the follow-up of recurrence and in an initial diagnosis setting in NMIBC and in comparison with routinely used screening methods such as cystoscopy and/or cytology. The first detected limitation of this study is the recurrence rate of only 28%. A value ranging 60–70% would be expected, as reported in the literature for NMINBC following TUR treatment (Antoine and van der Heijden, 2009; Kaufman et al., 2009; Chamie et al., 2013). The reason for this difference may reflect the restricted 2-year patients’ follow-up considered, or patient-related factors (age, gender, multiplicity, smoking status, and adjuvant treatment) associated with recurrence frequency that are not considered at the moment (Kobayashi et al., 2014).

The sensitivity and specificity of Uromonitor^®^ assay in the detection of TUR confirmed recurrence in the follow-up series are comparable to cystoscopy performance and in accordance with the literature that describes that about 20% of primary tumors lack *TERTp* and *FGFR3* alterations, rendering an empirical detection rate around 80%. The unsolved 20% of cases can be attributed to tumors and recurrences that may undergo different tumorigenic pathways other than the acquisition of *TERTp* and *FGFR3* alterations that could preclude Uromonitor^®^ testing capacity, like *RAS* mutations (see below).

Among the recurrence-negative cases, the test was concordantly negative in 87.7% of cases. The mutational state of *TERTp* and *FGFR3* genes in bladder cancer is considered a promising predictor of recurrence of NMIBC, demonstrated by the association between *FGFR3* mutation in primary tumor and later in recurrence events (Hernandez et al., 2006; Burger et al., 2008; Kompier et al., 2009; Miyake et al., 2010; Zuiverloon et al., 2010; van Rhijn et al., 2012). A specificity of 93.2% reflects the detection of three cases positive for *FGFR3* mutations and three cases with *TERTp* mutations without evidence of recurrence by cystoscopy. These cases remained negative for recurrence during the 2-year follow-up, suggesting that they were false positives. *TERTp* mutations were extensively studied and are described in the literature as absent in normal tissue. Since a clear positive signal was obtained in the aforementioned cases, we cannot rule out the hypothesis that Uromonitor^®^ high sensitivity may be detecting microscopic lesions and that may predict the appearance of a macroscopic lesion in a longer-term period beyond the 2-year follow-up.

More than half of the cases presented at least one mutational event, and it is reported that *TERTp* and *FGFR3* mutations tend to occur more frequently together than per chance; the combination of both constitutes a more reliable biomarker for NMIBC recurrence monitoring (Hosen et al., 2015; Critelli et al., 2016).

Uromonitor^®^ sensitivity performance was higher than cytology, and if used as an adjunct to cystoscopy it allowed achieving a 100% sensitivity and 88.6% specificity, an important upgrade in sensitivity and specificity in comparison to the cystoscopy and cytology combination. This data demonstrates that this test, used together with cystoscopy at a routine level, will lead to a cost-effectiveness increment. It can also be used *per se* without any decrease in performance relative to the current routinely used methods, such as cystoscopy or if cystoscopy is not routinely available.

Uromonitor^®^ showed overall good performance in recurrence detection across all stages. In Cis/Tis tumors, which represent 20% of follow-up positive cases, a peak performance was obtained with a 100% detection rate. For the majority of these Cis/Tis cases *TERTp* alterations were present (four out of five cases, 80.0%). Several authors reported that *TERTp* alterations are not associated with stage and *FGFR3* hotspot alterations are rare in Cis/Tis tumors; this is concordant with our findings where only one patient presented a *FGFR3* mutation.

This series was enriched with high-grade tumors, which is in contrast to the majority of the reported series of NMIBC. This might have created a bias in the results and impacted the assay performance. Development of this test is actively based in *TERTp*/*FGFR3* alterations detection that together are more frequent in low-grade tumors; additionally, *FGFR3* mutations are rare in high-grade tumors.

We also prospectively analyzed and compared initial diagnosis performance in comparison to routinely used screening methods. In an initial diagnosis setting, sensitivity was lower in the detection of disease compared to standard cystoscopy that is considered to virtually hold a 100% sensitivity (a value due to the absence of confirmation of tumor existence on cystoscopy negative cases) and 88.6% specificity. Among the negative cases, the Uromonitor^®^ test was concordant in 100% of cases being highly specific. In comparison with another routine assay, Uromonitor^®^ largely surpasses cytology’s sensitivity that could not detect any new bladder cancer-positive case (0% sensitivity).

Nevertheless, taking into consideration that Uromonitor^®^ main performance was initially aimed for low-grade tumors, these series gave us the opportunity to improve Uromonitor^®^ performance in a high-grade tumor-enriched setting. To this purpose, we included new biomarkers in the test in order to improve Uromonitor^®^ detection rate and testing capabilities overall; we want to achieve and maintain the high sensitivity and specificity that our test offers. This led to the *KRAS* hotspot alterations inclusion, and this preliminary data demonstrated that, both for follow-up or initial diagnosis cases, Uromonitor^®^ + *KRAS* improves significantly, reachin a 100% sensitivity in follow-up detection and 93.3% in initial-diagnosis detection and with an overall performance of 95.2% regardless of grade. With the inclusion of *KRAS* hotspot mutation screening together with Uromonitor^®^, this preliminary data presents this noninvasive approach as a true alternative to cystoscopy for NMIBC follow-up or even as a population screening and/or initial diagnosis for bladder cancer. It is also noteworthy to mention that the inclusion of *FGFR3* in Uromonitor^®^ test raises its usefulness as a biomarker test for targeted therapy.

During the course of this study, an interesting case demonstrated the capacities of these novel tests in providing new information regarding disease progression. In a patient apparently free of local disease, a *TERTp* mutation was detected in the urine. Although the patient did not present bladder cancer at the time, it had a hepatic metastasis. Further analysis confirmed that the hepatic metastasis presented a *TERTp* mutation. It will be worthwhile to investigate the usefulness of this test technology in the screening of other tumors and metastasis, namely, those harboring *TERTp* mutations, in urine samples.

In terms of the Uromonitor^®^ performance in comparison with other available options, it presented improved features. Reviewed by Sapre et al. (2014), the sensitivity of other available options ranges from 50.0% to 96.6%, and the tests are based on different methodology approaches, some more technically challenging and maintaining invasive requirements for the procedure. Avoidance of invasive procedures for the patients was a concern in the development of this test since morbidity of cystoscopy is often underestimated and can impact on patient adherence, with surveillance rates as low as 40% (Schrag et al., 2003). The fact that the test is conducted in urine renders it safer for patient use and with better acceptance in comparison with conventional cystoscopy. Another important aspect in the development of Uromonitor^®^ was the implementation ability across different centers or laboratories. For this, we concentrated on three pillars: ease of use, cost, and response time. Being a real-time PCR-based method, a technique that is already well implemented in most laboratories, not requiring a specialized technician to execute the test or dedicated apparatus, with affordable equipment and reduced costs, and, most determinately, with the capacity to output a result in 6 h. If we compare this approach with other established next-generation sequencing (NGS)-based methods, it is promptly detected that such a fast response is not possible as it is required to have a sample and library preparation, failing the short-time frame response, the costs would increase with run and equipment requirements and NGS equipment is not widely available. Also, if we compare this to current detection methods for *TERTp* and/or *FGFR3* based on Sanger sequencing, the high increase in sensitivity is a key factor, especially since we are trying to detect trace amount of tumor cells in urine samples.

Overall, this study demonstrates that Uromonitor^®^ represents a highly sensitive and specific urine test in detecting recurrence of NMIBC. Taking into account the obtained results, we can view Uromonitor^®^, with and without KRAS mutation screening, on different levels depending on the specific needs of the patient/healthcare professional. Uromonitor^®^ *TERTp*/*FGFR3* screening could be easily used in direct substitution of cytology since it presents an undoubtfully higher overall sensitivity while maintaining analysis response time and costs at an equivalent level. On the other hand, Uromonitor^®^ *TERT*/*FGFR3*/*KRAS* screening, based on our preliminary results, could directly substitute cystoscopy in a specific context, such as the impossibility of performing an cystoscopy, patients refusing to perform cystoscopy, or even alternating with cystoscopy in the follow-up program in patients with low-risk, low-grade lesions, alleviating the number of cystoscopy procedures that patients are required to undergo. The rate of Uromonitor^®^ false positives was similar to the rate of cystoscopy false positives. Our results prompt us to validate these findings in an enlarged robust independent series, in an ongoing study with a design that includes a group of benign conditions (renal lithiasis, urinary infections, hyperplasia of the prostate, and others). We intend to further test it and externally validate it to assess its cost-effectiveness and to determine its value in patients’ follow-up.

## Data Availability Statement

The raw data supporting the conclusions of this article will be made available by the authors, without undue reservation, to any qualified researcher.

## Ethics Statement

The studies involving human participants were reviewed and approved by Comissão de Ética do Instituto Português de Oncologia de Coimbra. The patients/participants provided their written informed consent to participate in this study.

## Author Contributions

RB performed all studies and wrote the draft of the manuscript. HP participated in the genetic analysis. JV, HP, VM, and PSo supervised the entire project and gave critical comments on the manuscript. CS participated in the genetic analysis, in the experimental design and managed the literature searches. PP, PC, AS, RL, AG, FF, CO, JT, PE, PA, FA, EG, BB, TE, PSt, AP, RA, AV, PB-V, NF, HÖ, CG-E, JM, TL, MA-M, PPS, SC, MP contributed to the sample and data collection for the study. All authors read and approved the final manuscript.

## Funding

This study was supported by FCT (“Portuguese Foundation for Science and Technology”) through a PhD grant to RB (SFRH/BD/111321/2015). Further funding was obtained from the project “Advancing cancer research: from basic knowledge to application” NORTE-01-0145-FEDER-000029: “Projetos Estruturados de I & D & I,” funded by Norte 2020—Programa Operacional Regional do Norte. This article is a result of the project PTDC/MED-ONC/31438/2017 (The Other Faces of Telomerase: Looking beyond Tumor Immortalization), supported by Norte Portugal Regional Operational Programme (NORTE 2020), under the PORTUGAL 2020 Partnership Agreement, through the European Regional Development Fund (ERDF), COMPETE 2020—Operacional Programme for Competitiveness and Internationalisation (POCI) and by Portuguese funds through FCT. Further funding by the European Regional Development Fund (ERDF) through the Operational Programme for Competitiveness and Internationalisation—COMPETE 2020, and Portuguese national funds *via* FCT, under project POCI-01-0145-FEDER-016390:CANCEL STEM.

## Conflict of Interest

RB, JV, HP, and PS are the founders of U-Monitor Lda, owner of the Uromonitor product. This company has licensed technologies from Ipatimup that are related to the work described in this paper (International Patent PCT/PT2016/050007—Method, sequences, compositions and kit for detection of mutations in the h gene promoter).

The remaining authors declare that the research was conducted in the absence of any commercial or financial relationships that could be construed as a potential conflict of interest.
