# Supplementary material for: Validation of a Novel, Sensitive, and Specific Urine-Based Test for Recurrence Surveillance of Patients With Non-Muscle-Invasive Bladder Cancer in a Comprehensive Multicenter Study
Source: Front Genet. 2019 Dec 18;10:1237. doi: 10.3389/fgene.2019.01237 (PMC6930177; doi:10.3389/fgene.2019.01237)
Supplement: Supplementary file 1 [file DataSheet_1.docx]

Supplementary Figures and Tables

Supplementary Table 1 – Samples inclusion and exclusion criteria for the study

| Inclusion criteria | Exclusion criteria |
| --- | --- |
| General urine samples study inclusion criteria:   - Adults ≥ 18 years of age - Patients with current or previous history of Bladder disease - Urine sample obtained immediately before cystoscopy procedure - Written informed consent acceptance | - **No written informed consent acceptance** - **Urine sample obtained after cystoscopy procedure** |
| Criteria for Eligibility for inclusion in technical validation analysis:   - Successfully characterized by Uromonitor Test | - **Unsuccessfully characterized for Uromonitor Test** |
| Criteria for Eligibility for inclusion in Follow-up clinical validation analysis:   - Complete patient information including cystoscopy Follow-up result - Patient diagnosed for NMIBC prior to cystoscopy and currently under Follow-up for NMIBC recurrence - Successfully characterized by Uromonitor Test | - **Unsuccessfully characterized for Uromonitor Test** - **Patient previously undiagnosed for NMIBC.** |
| Criteria for Eligibility for inclusion in Initial Diagnosis clinical validation analysis:   - Complete patient information including cystoscopy Follow-up result - Patient previously undiagnosed for NMIBC - Successfully characterized by Uromonitor Test | - **Unsuccessfully characterized for Uromonitor Test** - **Patient previously diagnosed for NMIBC prior to cystoscopy** |
| Criteria for Eligibility for inclusion in FFPE molecular characterization:   - FFPE block containing initial tumor or recidive tumor from NMIBC - Available at least two 10 µm cut from FFPE tissue - Material from adults ≥ 18 years of age - Written informed consent acceptance - Successfully characterized by Uromonitor Test | - **FFPE material does not contain tumor representativity** - **Not enough material or not enough quality is obtained from FFPE material** - **Unsuccessfully characterized for Uromonitor Test** - **No written informed consent acceptance** |

Supplementary Table 2 – Urology centres participant in this study

| COUNTRY | UROLOGY CANCER CENTER | URINE SAMPLES USED FOR MOLECULAR VALIDATION | URINE SAMPLES USED FOR CLINICAL VALIDATION | FFPE SAMPLES |
| --- | --- | --- | --- | --- |
| Germany | HELIOS Hospital, Department of Urology | 10 | 9 | 0 |
| Algeria | Algeria | 10 | 9 | 0 |
| Spain | Hospital Clinico San Carlos | 10 | 10 | 0 |
|  | Donostia – San Sebastian | 9 | 6 | 0 |
|  | Hospital Universitário de Araba | 10 | 10 | 0 |
|  | Hosp. Univ. Jimenez Dias | 7 | 7 | 0 |
|  | Farmadex | 30 | 17 | 0 |
| Portugal | IPO - Coimbra | 98 | 51 | 41 |
|  | CUF-Coimbra | 15 | 14 | 0 |
|  | Hospital Universidade Fernando Pessoa | 12 | 7 | 0 |
|  | Hospital Garcia de Horta | 10 | 10 | 0 |
|  | Centro Clinico Académico, Hospital de Braga | 49 | 12 | 0 |
|  | Hospital Santa Maria | 5 | 0 | 0 |
| Denmark | Sjaellands | 5 | 0 | 0 |
| Egypt | - | 19 | 0 | 0 |
| Netherlands | St. Antonius Ziekenhuis | 10 | 7 | 0 |
| Turkey | Gildron Saglik Teknolojileri Ithalat Ihracat | 9 | 7 | 0 |
| Ukraine | Ternopil Regional Oncology Clinic | 13 | 9 | 0 |

Supplementary Table 3 – Uromonitor® technical validation – accuracy

| ***Accuracy*** |  |  |  |  |  |
| --- | --- | --- | --- | --- | --- |
|  | ***-124 Assay*** | ***-146 Assay*** | ***FGFR3 248*** | ***FGFR3 249*** | ***Combined Assays*** |
| ***Urine samples*** | 100%  (n=109) | 98.6%  (n=108) | 87.3%  (n=91) | 94.2%  (n=88) | 95.0%  (n=396) |
| ***FFPE samples*** | 98.5%  (n=201) | 99.5%  (n=200) | 90.2%  (n=41) | 97.6%  (n=41) | 96.5%  (n=483) |

n = number of tests performed for the specific assay for accuracy calculation

Supplementary Table 4 – Different screening methods data in NMIBC Follow-up Recurrence detection and in NMIBC Initial Diagnosis

|  | |  | **FOLLOW-UP COHORT** | | |  | **INITIAL DIAGNOSIS COHORT** | | |
| --- | --- | --- | --- | --- | --- | --- | --- | --- | --- |
|  |  |  |  | | |  |  | | |
|  |  |  | ***Rec^+^*** | ***Rec^-^*** | ***Total*** |  | ***Ned^+^*** | ***Ned^-^*** | ***Total*** |
| **UROMONITOR** | ***Mut^+^*** |  | 25 | 6 | 31 |  | 14 | 0 | 14 |
|  | ***Mut^-^*** |  | 9 | 82 | 91 |  | 14 | 35 | 49 |
|  | ***Total*** |  | 34 | 88 | 122 |  | 28 | 35 | 63 |
|  |  |  |  | | |  |  | | |
|  |  |  | ***Rec^+^*** | ***Rec^-^*** | ***Total*** |  | ***Ned^+^*** | ***Ned^-^*** | ***Total*** |
| **CYSTOSCOPY** | ***Cys^+^*** |  | 27 | 6 | 33 |  | 28 | 4 | 14 |
|  | ***Cys^-^*** |  | 7 | 82 | 89 |  | 0 | 31 | 49 |
|  | ***Total*** |  | 34 | 88 | 122 |  | 28 | 35 | 63 |
|  |  |  |  | |  |  |  |  |  |
|  |  |  | ***Rec^+^*** | ***Rec^-^*** | ***Total*** |  | ***Ned^+^*** | ***Ned^-^*** | ***Total*** |
| **CYTOLOGY** | ***Cyt^+^*** |  | 6 | 2 | 8 |  | 0 | 4 | 4 |
|  | ***Cyt^-^*** |  | 8 | 31 | 39 |  | 7 | 26 | 33 |
|  | ***Total*** |  | 14 | 33 | 47 |  | 7 | 30 | 37 |
|  |  |  |  | |  |  |  |  |  |
|  |  |  | ***Rec^+^*** | ***Rec^-^*** | ***Total*** |  | ***Ned^+^*** | ***Ned^-^*** | ***Total*** |
| **CYSTOSCOPY**  **+**  **CYTOLOGY** | ***Cys/Cyt^+^*** |  | 13 | 4 | 17 |  | 7 | 4 | 11 |
|  | ***Cys/Cyt^-^*** |  | 2 | 29 | 31 |  | 0 | 26 | 26 |
|  | ***Total*** |  | 15 | 33 | 48 |  | 7 | 30 | 37 |
|  |  |  |  | |  |  |  |  |  |
|  |  |  | ***Rec^+^*** | ***Rec^-^*** | ***Total*** |  | ***Ned^+^*** | ***Ned^-^*** | ***Total*** |
| **UROMONITOR**  **+**  **CYSTOSCOPY** | ***Mut/Cys^+^*** |  | 34 | 12 | 46 |  | 28 | 4 | 32 |
|  | ***Mut/Cys^-^*** |  | 0 | 76 | 76 |  | 0 | 31 | 31 |
|  | ***Total*** |  | 34 | 88 | 122 |  | 28 | 35 | 63 |
|  |  |  |  |  |  |  |  |  |  |
|  |  |  | ***Rec^+^*** | ***Rec^-^*** | ***Total*** |  | ***Ned^+^*** | ***Ned^-^*** | ***Total*** |
| **UROMONITOR**  **+**  **KRAS** | ***Mut/G12^+^*** |  | 6 | 3 | 9 |  | 14 | 2 | 16 |
|  | ***Mut/G12^-^*** |  | 0 | 15 | 15 |  | 1 | 8 | 9 |
|  | ***Total*** |  | 6 | 18 | 24 |  | 15 | 10 | 25 |

Mut^+^ -Sample Positive for Uromonitor Test; Mut^-^ - Sample Negative for Uromonitor Test; Cys^+^ - Sample Positive for Cystoscopy; Cys^-^ -Sample Negative for Cystoscopy; Cyt^+^ - Sample Positive for Cytology; Cyt^-^ -Sample Negative for Cytology; Cys/Cyt^+^ - Sample Positive for Cystoscopy and/or Cytology; Cys/Cyt^-^ - Sample Negative for both Cystoscopy and Cytology; Mut/Cys^+^ - Sample Positive for Uromonitor test and/or Cystoscopy; Mut/Cys^-^ - Sample Negative for both Uromonitor test and Cystoscopy; Mut/G12^+^ - Sample positive for Uromonitor Test and/or KRAS screening; Mut/G12^-^ - Sample negative for both Uromonitor Test and KRAS screening.

Supplementary Table 5 - Cohort's tumor stage distribution

|  | Follow-Up Cohort | Initial Diagnosis Cohort |
| --- | --- | --- |
| Stage | Percentage of cases | Percentage of cases |
| Cis/Tis | 19.2% (5) | 0.0% (0) |
| Ta | 50.0% (13) | 76.0% (19) |
| T1 | 26.9% (7) | 20.0% (5) |
| T2 | 0.0% (0) | 4.0% (1) |
| Hep.Met | 3.8% (1) | 0.0% (0) |
| Total | 100,0% (26) | 100,0% (25) |

Supplementary Table 6 - Cohort's tumor grade distribution

|  | Follow-Up Cohort | initial Diagnosis Cohort |
| --- | --- | --- |
| Stage | Percentage of cases | Percentage of cases |
| Low-Grade | 33.3% (8) | 68.0% (17) |
| High-Grade | 66.7% (16) | 32.0% (8) |
| Total | 100,0% (24) | 100% (25) |

Supplementary Table 7 - Uromonitor performance in Recurrence detection across tumor stages

| Follow-up cohort | | | |
| --- | --- | --- | --- |
| Stage | Recurrence + cases | Uromonitor True + | Uromonitor False - |
|  | % of recurrence in cluster | Uromonitor Rate of detection | Uromonitor Rate of failure |
| Cis/Tis | 19.2% (5) | 100% (5) | 0% (0) |
| Ta | 50.0% (13) | 53.8% (7) | 46.2% (6) |
| T1 | 26.9% (7) | 71.4% (5) | 28.6% (2) |
| Hep.Met | 3.8% (1) | 100% (1) | 0% (0) |
| Total | 100% (26) | 69.2% (18) | 30.8% (8) |

Supplementary Table 8 - Uromonitor performance in Recurrence detection across tumor grade

| Follow-up cohort | | | |
| --- | --- | --- | --- |
| Grade | Recurrence + cases | Uromonitor True + | Uromonitor False - |
|  | % of recurrence in cluster | Uromonitor Rate of detection | Uromonitor Rate of failure |
| Low-Grade | 33.3% (8) | 62.5% (5) | 37.5% (3) |
| High-Grade | 66.7% (16) | 75.0% (12) | 25.0% (4) |
| Total | 100% (24) | 100% (17) | 29.2% (7) |


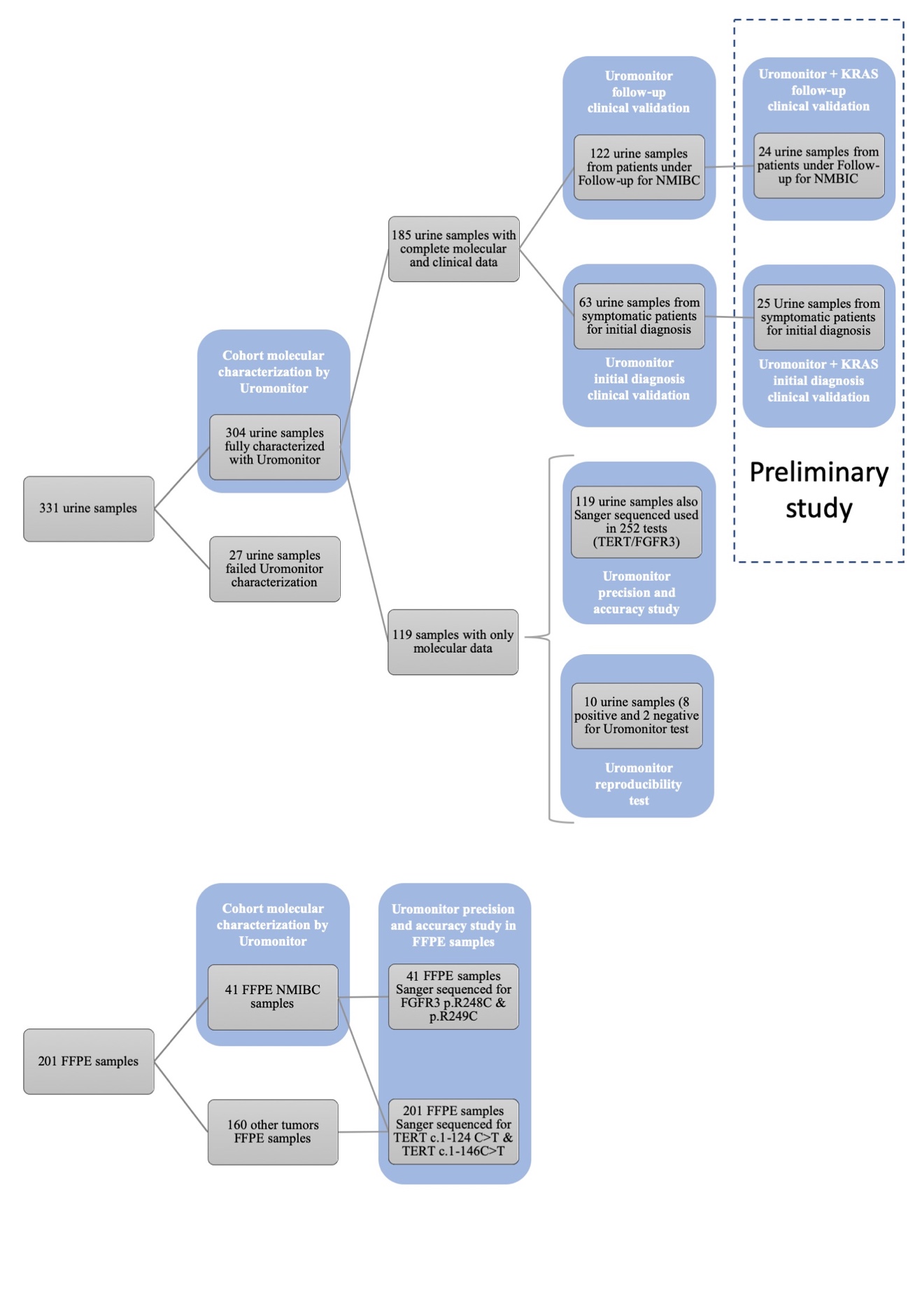


Supplementary Figure 1 - Study cohort's distribution and organization. Samples were subdivided according to the patient's donor material (Urine or FFPE) and used according to each study objective.


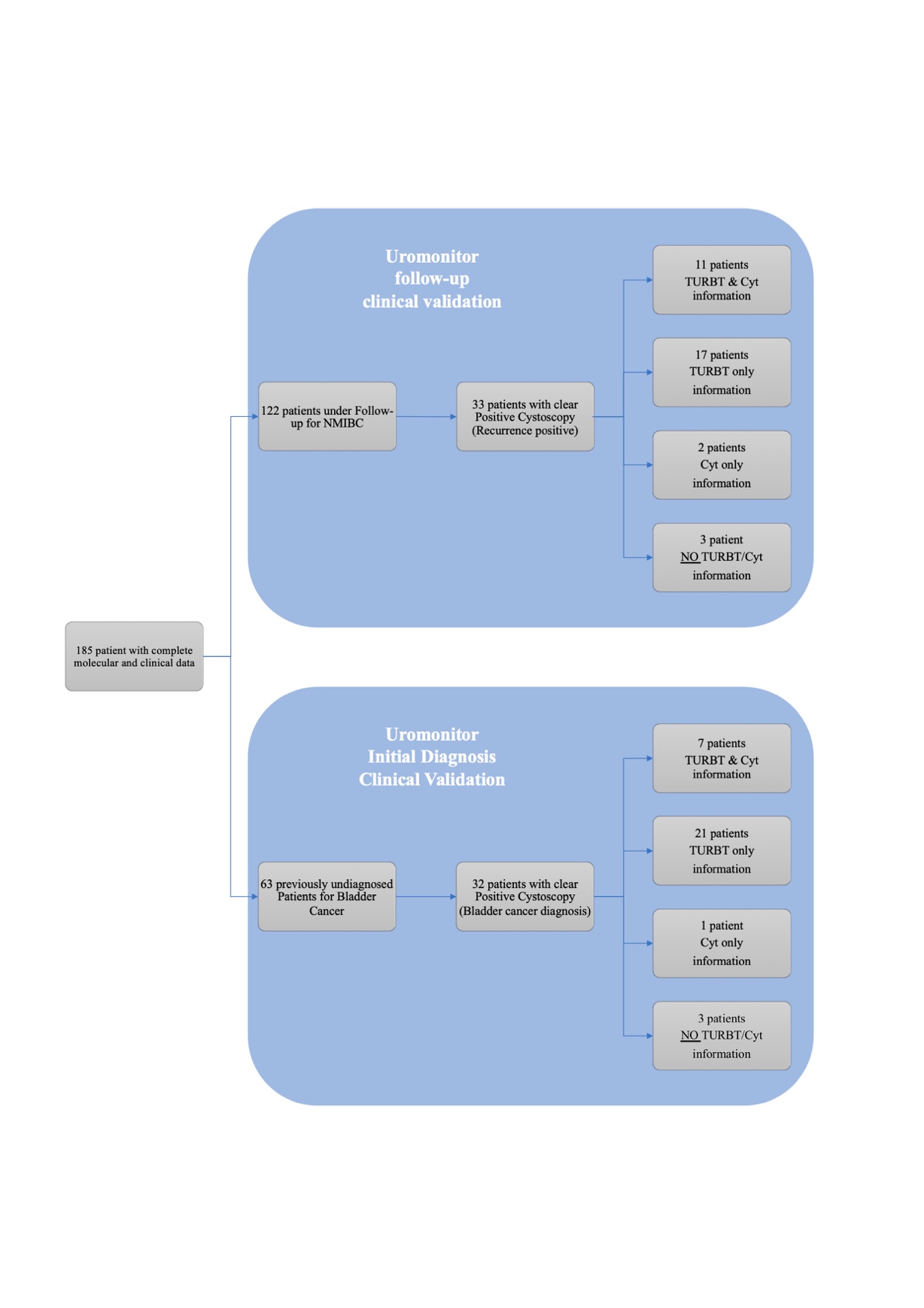


Supplementary Figure 2 – Patient’s Cystoscopy/Cytology data availability. Recurrence positive patients from each cohort could present TURBT biopsy and cytology information (TURBT & Cyt), only TURBT biopsy information (TURBT only), only cytology information (Cyt only) or no information on TURBT biopsy or cytology (NO TURBT/Cyt)


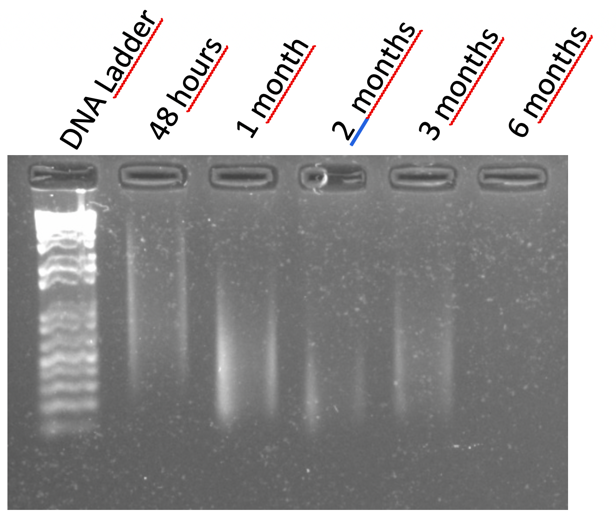


Supplementary Figure 3 – Qualitative DNA assessment on stored filter at 4ºC following nucleic acid extraction. One urine sample was filtered in 5 filters, stored at 4ºC. DNA was extracted 48h after filtering, up to 6 months on the filter. 100ng of total DNA was then run in a 0,8% agarose gel on 1X TBE buffer. High molecular weight quality DNA is obtained in filters stored up to 3 months at 4ºC


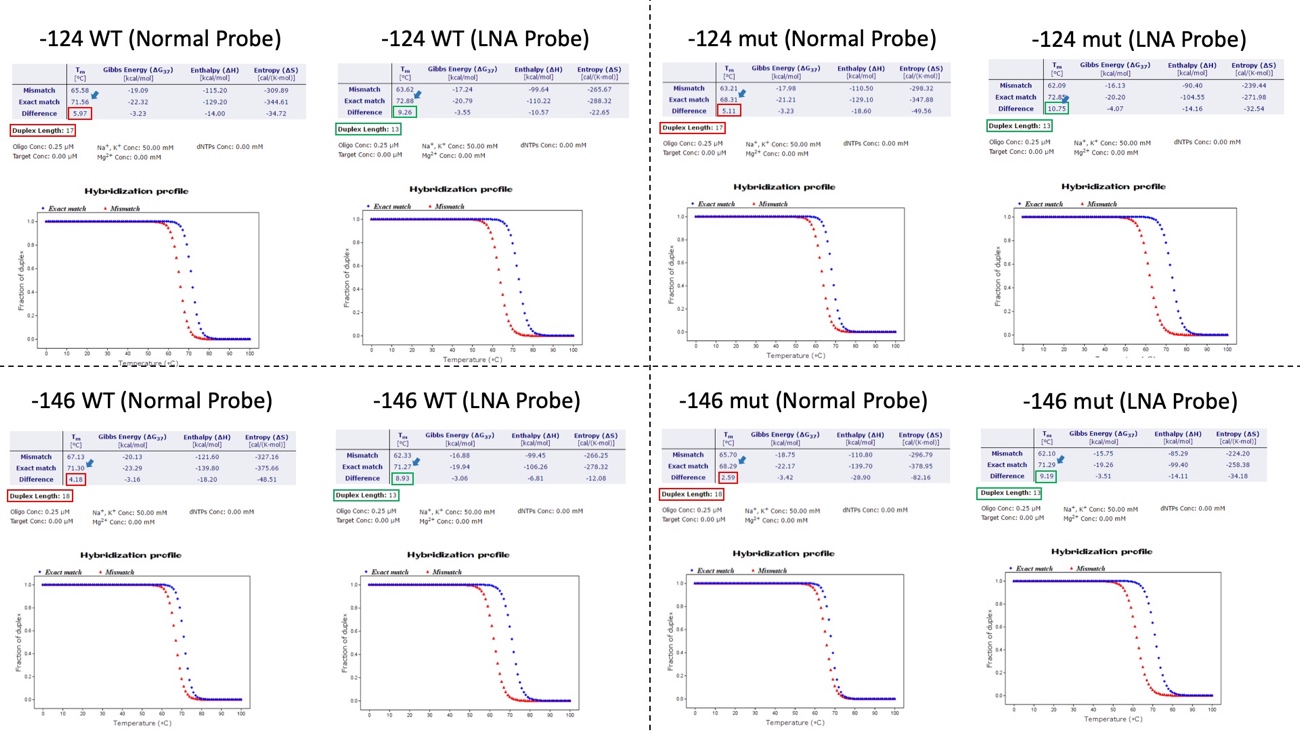
Supplementary Figure 4 – Probe design comparison (Normal probe VS LNA probe). A, B - Comparison between the design of regular fluorescent probes and LNA probes for both TERTp c.1-124C and c.1-124C>T sequences. The use of LNA bases in the probe structure allows to increase the overall melting temperature of the probe enhancing the possibility to achieve high melting temperatures with short probe sequences. This promotes specificity enhanced by a higher binding melting temperature difference on a presence of a base pair mismatch. C, D - Comparison between the design of regular fluorescent probes and LNA probes for both TERTp c.1-146C and c.1-146C>T sequences. The use of LNA bases in the probe structure allows to increase the overall melting temperature of the probe enhancing the possibility to achieve high melting temperatures with short probe sequences. This promotes specificity enhanced by a higher binding melting temperature difference on a presence of a base pair mismatch.

**D**

**B**

**A**

**C**

Supplementary Video 1 – <https://www.youtube.com/watch?v=UgnfL3-hH6Y>
